# Association of lifestyle, dietary pattern, and liver function with cognition in older adults: findings from a cross-sectional study

**DOI:** 10.3389/fnut.2025.1655601

**Published:** 2025-09-30

**Authors:** Xixiang Wang, Xiuwen Ren, Yu Liu, Lu Liu, Jingjing Xu, Shaobo Zhou, Ying Wang, Linhong Yuan

**Affiliations:** ^1^School of Public Health, Capital Medical University, Beijing Key Laboratory of Environment and Aging, China-British Joint Laboratory of Nutrition Prevention and Control of Chronic Diseases, Beijing, China; ^2^School of Science, Faculty of Engineering and Science, University of Greenwich, Chatham, United Kingdom; ^3^Suzhou Research Center of Medical School, Suzhou Hospital, Affiliated Hospital of Medical School, Nanjing University, Suzhou, China

**Keywords:** lifestyle, dietary pattern, liver function, mild cognitive impairment, AST/HDL-C

## Abstract

**Background:**

Single lifestyle, dietary pattern, and liver function are closely associated with cognitive ability, yet their combined influences on cognition remain unclear. This study aimed to investigate the effects of lifestyle, dietary patterns, and liver function on cognitive impairment among older adults.

**Methods:**

One thousand and ninety-six older adults were recruited from communities. Among them, 630 participants completed cognitive function tests. The lifestyle and dietary patterns of the participants were assessed using a healthy lifestyle score (HLS) and a healthy dietary score (HDS). Liver function was assessed using four predictive indicators: AST/HDL-C, ALT/HDL-C, HSI, and ZJU. Cognitive function was measured using the Montreal Cognitive Assessment (MoCA). Logistic regression, receiver operating characteristic curves (ROC), and restricted cubic splines (RCS) were applied to explore the relationship between variables.

**Results:**

A significant negative correlation was observed between HLS and liver function indicators (*r*_AST/HDL-C_ = −0.156, *r*_ZJU_ = −0.270, both *p* < 0.001), whereas a significant positive correlation was identified between HDS and MoCA scores (*r* = 0.074, *p* < 0.05). Poor liver function, represented by elevated plasma AST/HDL-C, was associated with increased mild cognitive impairment (MCI) risk (*OR* = 1.029, *p* = 0.007). ROC analysis showed that plasma AST/HDL-C had the highest predictive power for MCI (AUC = 0.634). RCS analysis revealed that AST/HDL-C and ALT/HDL-C were positively correlated with the risk of MCI, with cut-off values of 14.1 and 10.1, respectively.

**Conclusion:**

Impaired liver function is strongly associated with cognitive impairment, highlighting the critical role of maintaining healthy liver function in preventing MCI in the elderly. A healthy lifestyle positively correlated with both liver and cognitive functions, and a balanced diet significantly improved cognitive outcomes.

## 1 Introduction

Mild cognitive impairment (MCI) is an intermediate state between normal aging and dementia, such as Alzheimer’s disease (AD), and is characterized by a loss of memory and a decline in learning and cognitive function (1). Studies have shown that several years before the onset of AD, metabolic abnormality can be observed in blood biochemical markers related to the disease (2), such as increased blood β-amyloid (Aβ), tau, and neurofilament light chain (NfL) levels. *In vivo* studies also reported the signals and substances that communicate between the liver and brain through the nervous and circulatory systems; thereby, the concept of the ‘liver-brain axis’ was raised accordingly (3). Data from numerous animal experiments have explored the linkage of liver function with cognition from the biological perspective (4, 5), further demonstrating the impact of the liver-brain axis on cognitive function, including alteration of the blood–brain barrier (BBB) structure (6), generation of toxic metabolites (7, 8), and disruption in normal Aβ metabolism (5).

Various prediction models have been proposed to evaluate liver function, such as the fatty liver index (FLI) (9), hepatic steatosis index (HSI) (10), AST/HDL-C and ALT/HDL-C (11), and elevation of these indicators may partially indicate a decline in liver function. Moreover, the relation between damaged liver function and cognitive impairment has also been established in a population-based study (12–14). Therefore, improving cognition by enhancing liver function may represent a novel strategy for preventing cognitive dysfunction in the elderly.

The American Heart Association recommends adopting a healthy lifestyle and balanced dietary pattern to promote overall health (15). Smoking (16) and alcohol consumption (17) are well-established risk factors for numerous chronic diseases. In contrast, several studies have shown that tea consumption (18, 19) and regular physical activity (20, 21) benefit both liver and cognitive functions. Studies have found that older adults who remain physically active tend to preserve cognitive abilities for a longer period compared with their sedentary counterparts (22). Moreover, sleep disorders and insufficient sleep duration have been identified as important risk factors for both neurocognitive decline and liver disease (21, 23). Collectively, these findings suggest that a healthy lifestyle—including non-smoking, moderate alcohol use, regular physical activity, and adequate sleep—plays a pivotal role in maintaining liver and brain health. Moreover, adherence to balanced dietary patterns is equally critical for promoting overall health. In addition to a healthy lifestyle, the role of balanced dietary patterns in promoting body health has been extensively reported. The Chinese Food Guide Pagoda, developed based on the Dietary Guidelines for Chinese Residents, also encourages Chinese residents to consume diverse food items to ensure adequate nutrient intake and mitigate the risk of chronic diseases (24).

Many studies have explored the relationship between a single lifestyle, dietary intake, and liver function with cognitive function in the elderly, respectively. However, the combined impacts of lifestyle and dietary patterns on liver and cognitive functions remain unclear. This cross-sectional study was designed to examine how lifestyle and dietary patterns affect liver and cognitive functions in the elderly and whether there are synergistic effects. In addition, given the increasing recognition of liver function indicators as potential biomarkers, it is important to explore their predictive ability for mild cognitive impairment. Therefore, this study also aimed to evaluate whether liver function indicators could predict the risk of MCI by applying receiver operating characteristic (ROC) curve analysis.

## 2 Materials and methods

### 2.1 Study population

This study was designed as a community-based cross-sectional study. Specifically, from 2017 to 2023, a total of 1,096 community-dwelling older adults were initially recruited from the Nanyuan, Wulituo, and Guang’anmen communities in Beijing, as well as from Suzhou Science and Technology City Hospital in Suzhou. All participants completed blood sampling and surveys on dietary intake and lifestyle. Six hundred thirty participants also underwent the cognitive assessment (MoCA). Accordingly, analyses of diet, lifestyle, and liver function were conducted using data from all 1,096 participants, whereas analyses involving cognitive function were restricted to the 630 participants with complete cognitive data. Thus, the 630 participants represent a subset of the overall population. Participants were categorized based on cognitive status: those meeting the criteria for MCI were defined as the MCI group, while participants who did not meet the MCI criteria were considered the control group. A detailed flow chart of participant inclusion, including recruitment years, is presented in Supplementary Figure 1. All study procedures adhered to the ethical standards of the Helsinki Declaration of 1975, and written informed consent were obtained from all participants.

### 2.2 Inclusion and exclusion criteria

Participants were included if they: (1) were community-dwelling older adults aged ≥ 60 years; (2) completed both liver function assessments and cognitive function evaluation; and (3) provided complete information on lifestyle and dietary intake. Exclusion criteria were: (1) history of severe psychiatric disorders or neurodegenerative diseases other than MCI; (2) missing key demographic, biochemical, or cognitive data; and (3) inability to provide informed consent.

### 2.3 Demographic characteristics

Participants underwent a general information survey, medical history assessment, and physical examination. Information on demographic characteristics (age, gender, and education duration) and medical history of chronic diseases (hyperlipidemia, kidney disease, and type 2 diabetes mellitus (T2DM)) was collected through a self-administered questionnaire. Height and body weight were measured by trained nurses at the community medical service center. Body mass index (BMI) was calculated as weight (kg)/height squared (m^2^).

### 2.4 Lifestyle survey and healthy lifestyle score

The lifestyle variables included living alone, smoking, alcohol consumption, tea drinking, reading, TV/computer use, housework, physical activity, and dietary supplement use. Participants provided yes/no responses for these variables. The healthy lifestyle score (HLS) was developed according to findings from previous studies (Supplementary Table 1). Specifically, five lifestyle items, including non-smoking, non-drinking, tea consumption, engaging in housework, and weekly regular physical exercise, were assigned values for calculating the HLS. The total HLS score was calculated as the number of criteria met by each participant. The five items included in the HLS calculation have been indicated to link to both liver and cognitive functions (25–30). During data analysis, the HLS was further categorized into Low (1–2), Middle (3), and High (4–5).

### 2.5 Dietary survey and healthy dietary score

The participants’ daily food intake was obtained using a food frequency questionnaire (FFQ), including food consumption frequency (daily or weekly) and consumption quantity. The assessed food items included cereals, vegetables, fruits, animal food, milk and dairy products, soybeans and nuts, and cooking oil. The dietary survey was conducted by trained nurses from the community health center. The healthy dietary score (HDS) was calculated according to compliance with the Chinese Food Guide Pagoda (2022) (Supplementary Table 2). Specifically, for each food category, if the recommended daily intake (g/day) was met, a score of ‘1’ was assigned; otherwise, a score of ‘0’ was given. The total HDS was calculated as the sum of all scores across food categories for each individual. During data analysis, the HDS was categorized into Low (0–2) and High (3–4).

### 2.6 Biochemical measurements and evaluation of liver function

Fasting venous blood (5 mL) was collected from each participant in the morning and centrifuged at 480 g for 20 min to separate plasma. The separated plasma measured biochemical parameter measurement. Plasma glucose (Glu), total cholesterol (TC), and triglycerides (TG) were measured using the ILAB600 clinical chemistry analyzer (Instrumentation Laboratory, Lexington, WI, United States). High-density lipoprotein cholesterol (HDL-C) was measured using a commercially available assay from the Instrumentation Laboratory, and low-density lipoprotein cholesterol (LDL-C) was calculated according to the Friedewald formula. Plasma levels of alanine aminotransferase (ALT) and aspartate aminotransferase (AST) were measured using an autoanalyzer (COBAS Mira). All samples for each participant were analyzed within a single batch, and the inter-assay coefficient of variation (CV) was below 5%. Liver function was assessed using specific predictors reported by previous studies (10, 11, 31). To comprehensively evaluate liver function, we selected four commonly used indicators based on previous literature: HSI, AST/HDL-C, ALT/HDL-C, and ZJU. AST/HDL-C and ALT/HDL-C combine liver enzyme measurements with lipid metabolism parameters, providing a more integrative assessment of liver function in relation to systemic metabolic status. HSI and ZJU index are validated non-invasive predictors of fatty liver and overall liver health in population studies. Together, these four indicators capture both enzyme-based and non-enzyme-based aspects of liver function, allowing a more nuanced evaluation of liver status and its potential association with cognitive outcomes. The formulas for these predictors, including AST/HDL-C, ALT/HDL-C, HSI, and ZJU, are provided in Supplementary Table 3.

### 2.7 Evaluation of cognitive function

The MoCA scale was applied to evaluate participants’ cognitive function. The test was carried out by nurses and doctors trained uniformly at the community health service center. Based on findings from cognitive function screening in elderly Chinese populations (32), the MoCA score cut-off values for diagnosing MCI were as follows: 13/14 for illiterate individuals, 19/20 for those who received up to 6 years of education, and 24/25 for those received more than 6 years of education.

### 2.8 Statistical analysis

Statistical analyses were performed using IBM SPSS 23.0 and R 4.0.3. Continuous variables were expressed as mean ± standard deviation (SD), and differences between groups were analyzed using the Student’s *t*-test or the Mann–Whitney *U* test. Categorical variables were expressed as n (%), and group differences were analyzed using the chi-square test. Pearson or Kendall correlation coefficients were used to analyze the relationships between indicators. Logistic regression was performed to analyze the effects on the risk of MCI associated with different levels of HLS, HDS, and various liver function indicators. Calibration curves and ROC curves of the prediction model were plotted to evaluate its diagnostic value for predicting MCI. Potential non-linear associations were also explored using restricted cubic splines (RCS) with three knots for the indicators. A *p*-value < 0.05 was considered statistically significant.

## 3 Results

### 3.1 Demographic characteristics

The results for all participants (*N* = 1,096) are presented in Supplementary Table 4. The HDS for the general population ranged from 1 to 6 (Cooking oil data missing), with a median value of 1. The HLS ranged from 1 to 5, with a median of 4. The results of participants’ complete cognitive measurement (*N* = 630) are provided in Table 1. Patients with MCI had lower MoCA scores and shorter education duration (*p* < 0.05). In terms of lifestyle, MCI patients were more likely to drink tea and less likely to engage in reading or housework (*p* < 0.05). Regarding liver function, MCI patients had a higher plasma AST/HDL-C ratio (*p* < 0.05). There were no significant differences in HDS or HLS between groups (*p* > 0.05).

**Table 1:** Comparison of demographic characteristics, dietary intake, and plasma biochemical indices between MCI and control subjects (*N* = 630).

Variables	MCI (*N* = 330)	Control (*N* = 300)	*P*-value
Demographic characteristics			
Age, year (mean ± SD)*	69.13 ± 6.17	68.84 ± 4.87	0.506
Sex [male (%)]^†^	184 (55.8)	171 (57.0)	0.753
BMI (kg/m^2^)*	24.05 ± 3.33	23.81 ± 3.31	0.365
MoCA	19.37 ± 4.99	25.13 ± 2.93	**<0.001**
Education duration			**0.011**
≤9 years	231 (70.0)	181 (60.3)	
>9 years	99 (30.0)	119 (39.7)	
Hyperlipidemia (yes), [*n* (%)]	133 (40.3)	123 (41.0)	0.859
Kidney disease (yes), [*n* (%)]	31 (9.4)	23 (7.7)	0.439
T2DM (yes), [*n* (%)]	94 (28.5)	77 (25.7)	0.427
Lifestyle, *n* (%)^†^			
Living alone (yes)	24 (7.3)	21 (7.0)	0.894
Smoking (yes)	50 (15.2)	44 (14.7)	0.856
Alcohol drinking (yes)	93 (28.2)	85 (28.3)	0.966
Tea drinking (yes)	195 (59.1)	153 (51.0)	**0.041**
Reading (yes)	99 (30.0)	123 (41.0)	**0.004**
TV and computer (yes)	315 (95.5)	288 (96.0)	0.736
House working (yes)	289 (87.6)	288 (96.0)	**<0.001**
Physical activity (yes)	64 (19.4)	62 (20.7)	0.690
Dietary supplement (yes)	92 (27.9)	66 (22.0)	0.089
Dietary intake, (g/d)§			
Cereal	305.36 (219.64, 408.93)	305.36 (238.39, 404.93)	0.973
Vegetable	275.00 (225.00, 500.00)	275.00 (175.00, 525.00)	0.080
Fruit	125.00 (75.00, 225.00)	125.00 (75.00, 225.00)	0.997
Animal food	117.86 (76.79, 169.64)	107.14 (75.00, 151.79)	0.175
Soybean and nut	53.57 (26.79, 90.18)	50.00 (26.79, 78.57)	0.146
Milk	125.00 (42.86, 237.05)	114.29 (21.43, 214.29)	0.608
Cooking oil	27.60 (16.91, 38.33)	25.56 (15.33, 38.33)	0.521
Plasma biochemical indices (mmol/L)*			
Glu	6.05 ± 1.80	5.83 ± 1.61	0.104
TC	5.03 ± 1.04	5.07 ± 1.10	0.687
TG	1.63 ± 1.00	1.62 ± 1.19	0.883
LDL-C	3.04 ± 0.89	3.13 ± 0.96	0.212
HDL-C	1.41 ± 0.29	1.43 ± 0.31	0.462
Liver function^§^			
AST (IU/L)	20.00 (15.40, 25.00)	19.00 (13.12, 23.00)	0.075
ALT (IU/L)	16.00 (11.00, 22.56)	15.00 (8.99, 21.00)	0.146
ALT/HDL-C	11.43 (7.50, 17.05)	10.14 (5.94,14.98)	0.175
AST/HDL-C	14.92 (10.73, 19.09)	13.38 (9.32, 17.25)	**0.021**
HSI	32.15 (28.75, 36.07)	31.83 (28.15, 36.54)	0.811
ZJU	35.02 (31.69, 38.98)	34.64 (31.35, 38.00)	0.577
Comprehensive evaluation^§^			
HDS	1.00 (0.00, 2.00)	1.00 (0.00, 2.00)	0.738
HLS	3.00 (3.00, 4.00)	3.00 (3.00, 4.00)	0.308

The data were expressed as *M* (SD) or *n* (%) according to whether the continuous or category variable, respectively. MCI, mild cognitive impairment; BMI, body mass index; MoCA, Montreal cognitive assessment; T2DM, type 2 diabetes mellitus; Glu, glucose; TC, total cholesterol; TG, triglyceride; HDL-C, high-density lipoprotein cholesterol; LDL-C, low-density lipoprotein cholesterol; AST, aspartate transaminase; ALT, alanine aminotransferase; HSI, hepatic steatosis index; HDS, healthy diet score; HLS, healthy lifestyle score. *Student *t*-test was used; ^†^Chi-square test was used; ^§^Mann–Whitney *U* test was used. *P* < 0.05 is considered statistically significant and is represented in the table by bold numbers.

### 3.2 Correlation between HDS, HLS, and liver and cognitive function

**Figure 1:**
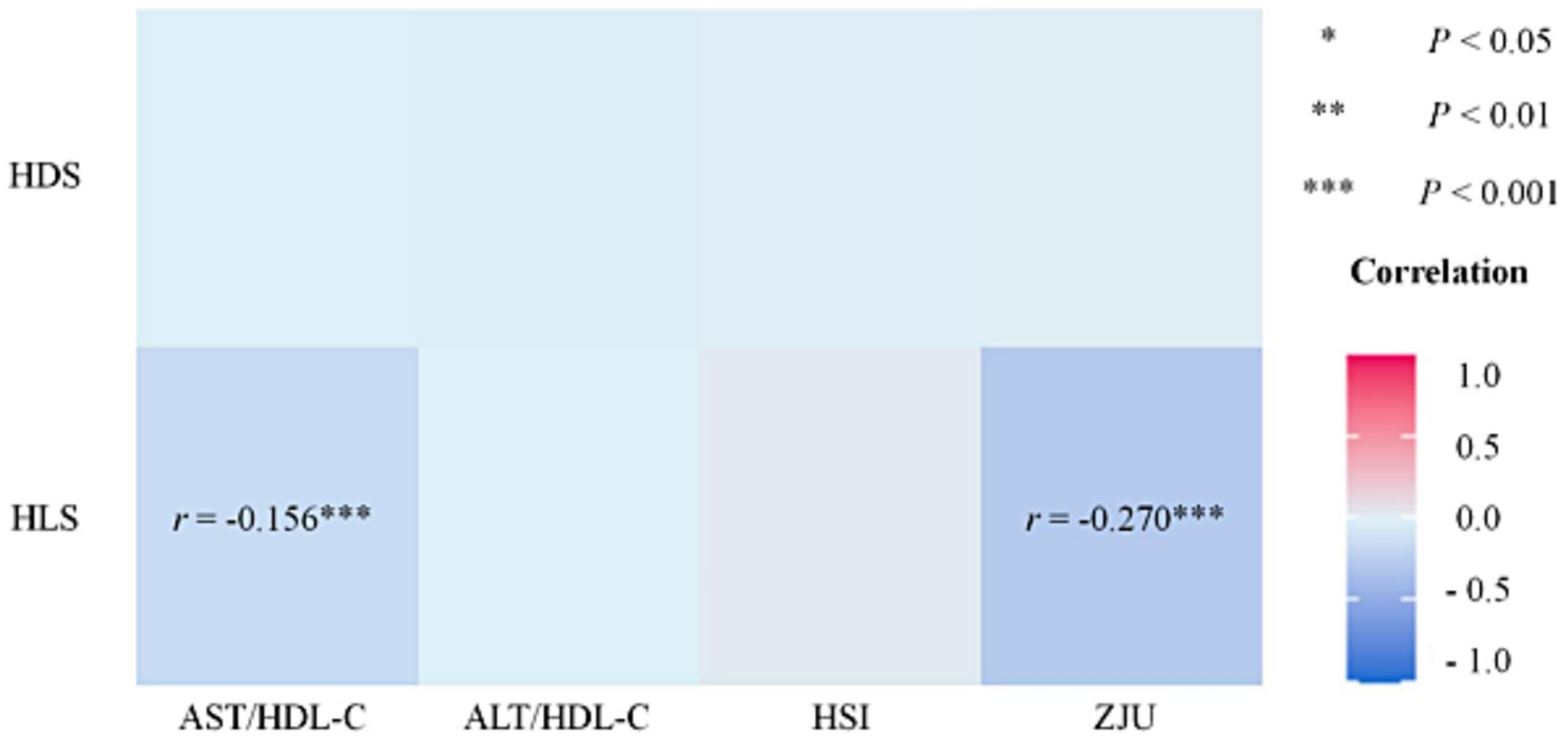
Correlation of HDS, LDS, and liver function (*N* = 1,096). HDS, healthy diet score; HLS, healthy lifestyle score; AST, aspartate transaminase; ALT, alanine aminotransferase; HDL-C, high-density lipoprotein cholesterol; HSI, hepatic steatosis index. *p < 0.05, **p < 0.01, ***p < 0.001.

The correlation results for total participants (*N* = 1,096) regarding diet, lifestyle, and liver function are presented in Figure 1. There was a significant negative correlation between AST/HDL-C with ZJU and HLS (*r*_AST/HDL-C_ = −0.156, *r*_ZJU_ = −0.270, both *p* < 0.001).

**Figure 2:**
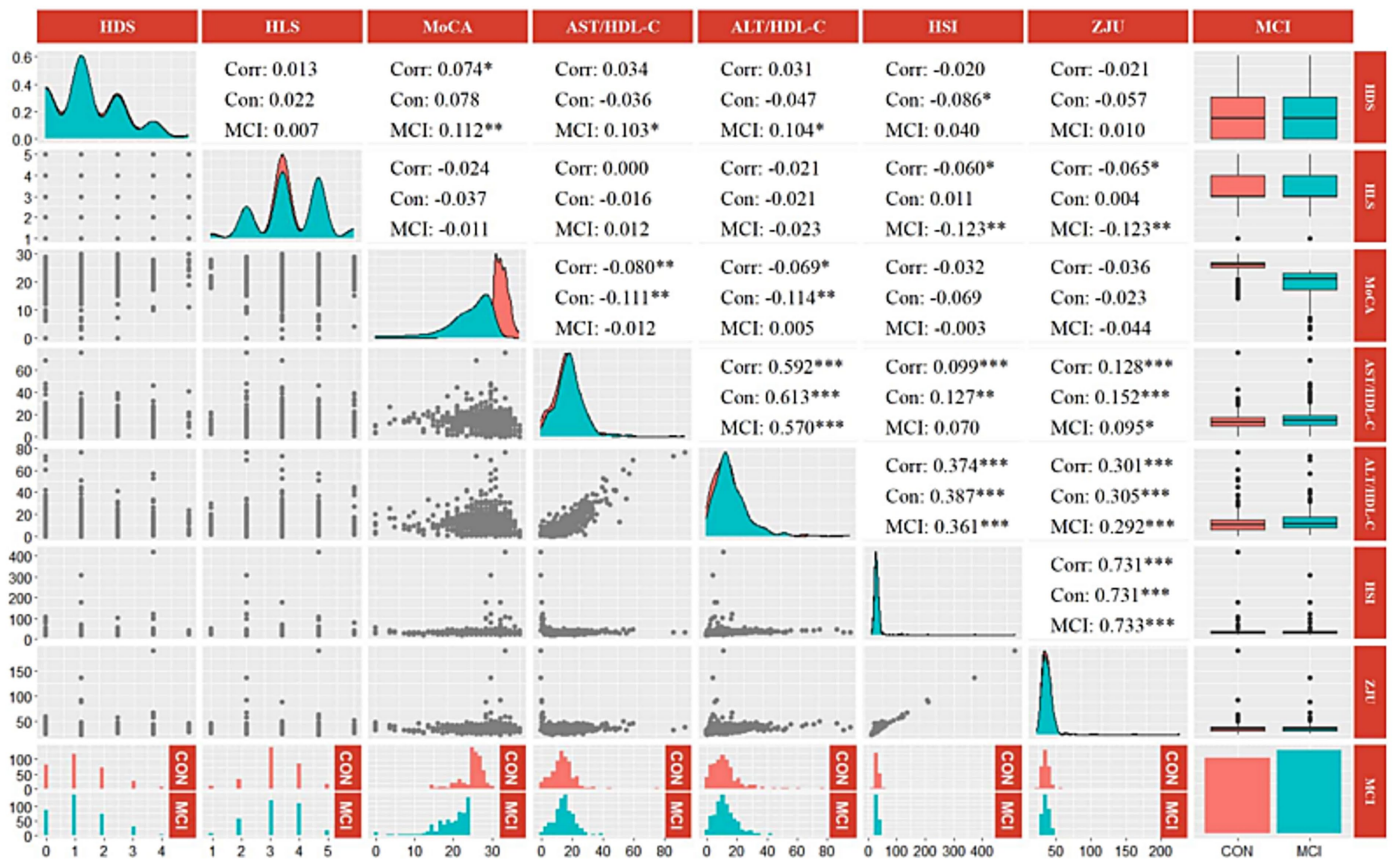
Correlation heatmap between HDS, HLS, MoCA score, and liver function indicators (AST/HDL-C, ALT/HDL-C, HSI, and ZJU) among different groups. HDS, healthy diet score; HLS, healthy lifestyle score; MoCA, Montreal cognitive assessment; AST, aspartate transaminase; ALT, alanine aminotransferase; HDL-C, high-density lipoprotein cholesterol; HSI, hepatic steatosis index. *p < 0.05, **p < 0.01, ***p < 0.001.

The correlation results for participants with cognitive outcomes (*N* = 630) regarding diet, lifestyle, liver, and cognitive function are illustrated in Figure 2. There was a significantly positive correlation between HDS and MoCA (*r* = 0.074, *p* < 0.05), as well as a significantly negative correlation between HLS with ZJU and HSI (*r*_HSI_ = −0.060, *r*_ZJU_ = −0.065, both *p* < 0.05). MoCA score was negatively correlated with AST/HDL-C and ALT/HDL-C (*r*_AST/HDL-C_ = −0.080, *r*_ALT/HDL-C_ = −0.069, both *p* < 0.05). There is a significant negative correlation between the HLS and liver function indicators (*r*_HSI_ = −0.123, *r*_ZJU_ = −0.123, both *p* < 0.01) in the MCI population; while in the control group, there is a significant negative correlation between liver function indicators and cognitive scores (*r*_AST/HDL-C_ = −0.111, *r*_ALT/HDL-C_ = −0.114, both *p* < 0.01). Significantly positive correlations were observed among the four liver function indexes (*p* < 0.05). However, there was no significant correlation between HDS and the liver function indexes (AST/HDL-C, ALT/HDL-C, HSI, and ZJU) in either the total participants (*N* = 1,096) or those with cognitive outcomes (*N* = 630).

### 3.3 Association between HDS, HLS, and liver and cognitive function

The association of HDS, HLS, and liver function indicators with the risk of MCI is presented in Table 2. No relationship was observed between HDS and the risk of MCI. However, a significantly negative correlation was detected between HLS and the risk of MCI. Individuals with a middle HLS level (compared to those with a low HLS of 1–2) exhibited a significantly decreased risk of MCI (*OR* = 0.535, *p* = 0.006). After adjusting covariates, the protective role of HLS in decreasing the risk of MCI was consistently observed (*OR* = 0.500, *p* = 0.003). In terms of liver function, after adjusting for covariates, a positive correlation between AST/HDL-C or ALT/HDL-C levels with the risk of MCI was detected (*OR*_AST/HDL_ = 1.029, *p* = 0.007; *OR*_ALT/HDL_ = 1.015, *p* = 0.088). However, no significant association was observed between HSI, ZJU, and the risk of MCI.

**Table 2:** Association of HDS, HLS, and liver function with the risk of MCI.

Variable	*N*	Logistic regression, OR (95% CI)			
		Unadjusted model	*p*-value	Adjusted model	*P*-value
HDS					
Low (0–2)	421	Reference		Reference	
High (3–4)	209	0.931 (0.668, 1.298)	0.675	0.960 (0.687, 1.343)	0.813
HLS					
Low (1–2)	112	Reference		Reference	
Middle (3)	275	0.535 (0.341, 0.837)	**0.006**	0.500 (0.317, 0.789)	**0.003**
High (4–5)	243	0.766 (0.485, 1.210)	0.253	0.769 (0.484, 1.221)	0.265
Liver function					
AST/HDL-C	630	0.963 (0.917, 1.011)	0.127	1.029 (1.008, 1.051)	**0.007**
ALT/HDL-C	630	0.985 (0.929, 1.044)	0.608	1.015 (0.998, 1.033)	0.088
HSI	630	0.999 (0.992, 1.006)	0.742	0.998 (0.991, 1.006)	0.657
ZJU	630	1.002 (0.985, 1.018)	0.840	1.000 (0.984, 1.017)	0.969

The association between variables (HDS, HLS, and liver function) and the risk of MCI was determined by logistic regression (*n* = 630). Model 1 was a crude model; model 2 was adjusted for education duration and reading. MCI, mild cognitive impairment; HDS, healthy diet score; HLS, healthy lifestyle score; AST, aspartate transaminase; ALT, alanine aminotransferase; HDL-c, high-density lipoprotein cholesterol; HSI, hepatic steatosis index. *P* < 0.05 is considered statistically significant and is represented in the table by bold numbers.

### 3.4 Comparison of diagnostic models

**Figure 3:**
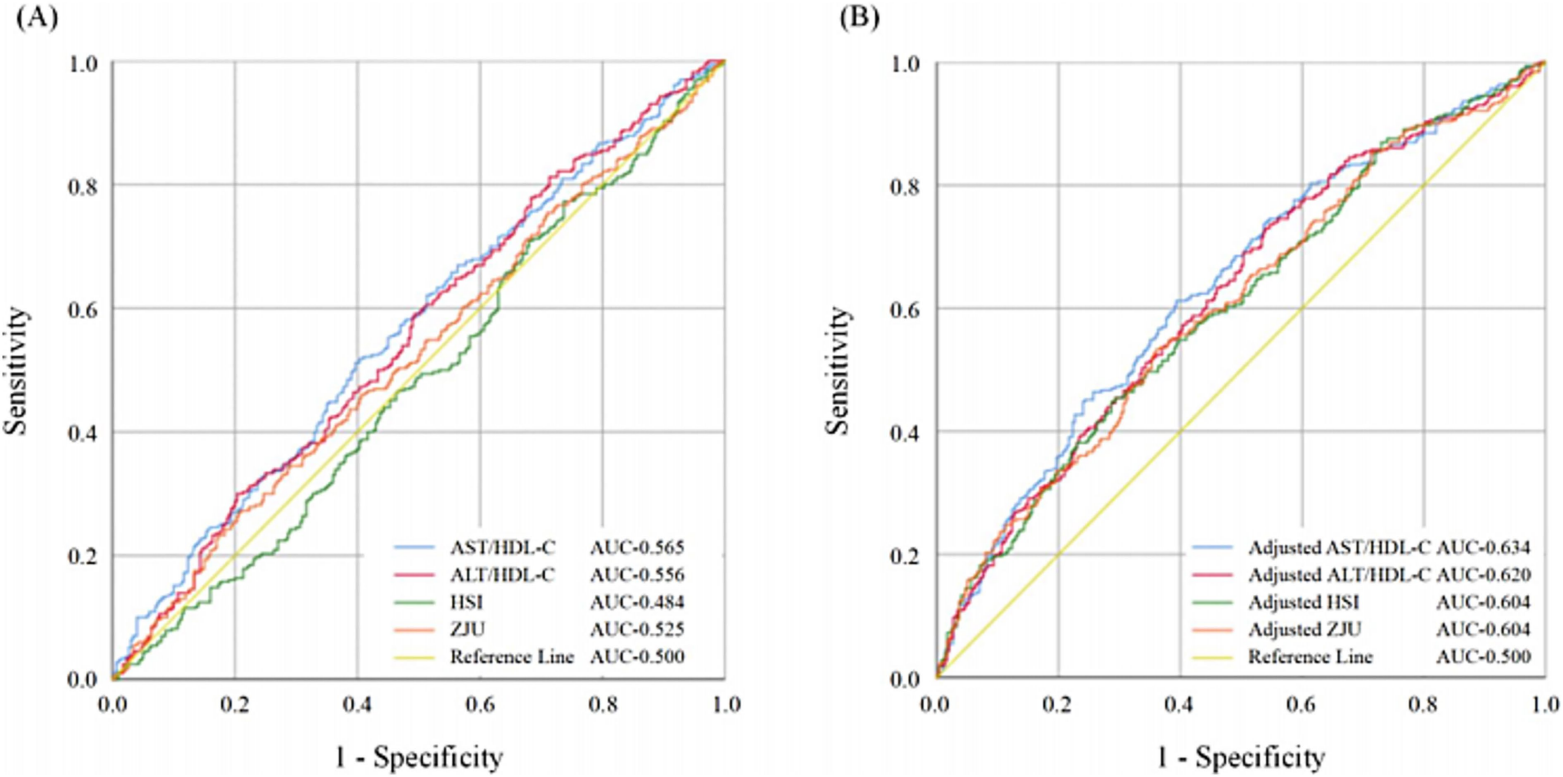
The ROC curves of the models were constructed based on AST/HDL-C, ALT/HDL-C, HSI, and ZJU. Comparison of ROC curves **(A)** without adjustment of confounding factors; **(B)** with adjustment of confounding factors including HDS, HLS, education duration, and reading. MCI, mild cognitive impairment; HDS, healthy diet score; HLS, healthy lifestyle score; AST, aspartate transaminase; ALT, alanine aminotransferase; HDL-C, high-density lipoprotein cholesterol; HSI, hepatic steatosis index.

The ROC curves of models under different adjustment conditions are presented in Figure 3, and the diagnostic value of the models was compared. In Model 1 (Figure 3A), no covariates were adjusted. In Figure 3B, Model 2 included adjustments for HDS, HLS, education duration, and reading. The AUC of the ROC curve for the model constructed based on AST/HDL-C was higher than those of Model 1 and Model 2 (AUC_Model1-AST/HDL-C_ = 0.565, AUC_Model2-AST/HDL-C_ = 0.634).

### 3.5 RCS between liver function and MCI

**Figure 4:**
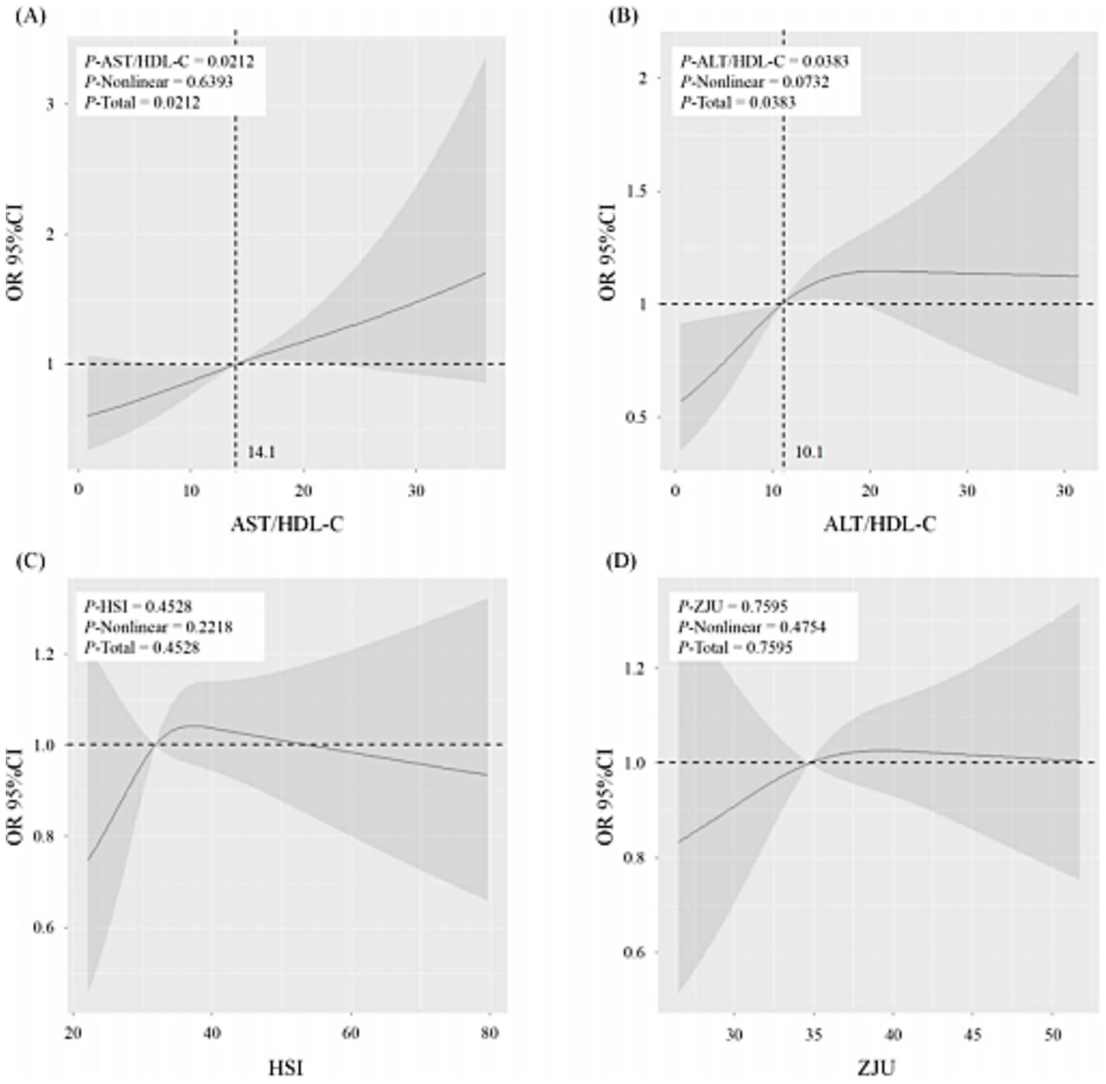
The restricted cubic spline (RCS) of the association between liver function (AST/HDL-C **(A)**, ALT/HDL-C **(B)**, HSI **(C)**, and ZJU **(D)**) and the risk of MCI. The solid lines indicate estimates of the risk of MCI across the continuous level of liver function indices (AST/HDL-C, ALT/HDL-C, HSI, and ZJU), fitted using logistic regression analysis. The shaded areas indicate the 95% confidence intervals. MCI, mild cognitive impairment; AST, aspartate transaminase; ALT, alanine aminotransferase; HDL-c, high-density lipoprotein cholesterol; HSI, hepatic steatosis index. *p* < 0.05 was considered statistically different.

In Figure 4, the RCS demonstrated a dose–response relation between liver function indicators (AST/HDL-C, ALT/HDL-C, HSI, and ZJU) and the risk of MCI. The AST/HDL-C and ALT/HDL-C were positively correlated with the risk of MCI in the absence of non-linear associations (*P*-AST/HDL-C = 0.0212, *P*-ALT/HDL-C = 0.0383, both *P*-Non-linear > 0.05) (Figures 4A,B). The cut-off values for AST/HDL-C and ALT/HDL-C were 14.1 and 10.1, respectively. However, HSI and ZJU were not significantly associated with the risk of MCI (*p* > 0.05).

## 4 Discussion

In this study, we constructed a HLS and a HDS based on an individual’s lifestyle and dietary pattern to comprehensively assess their individual effects on liver and brain function. We found that HLS was significantly negatively correlated with AST/HDL-C and ZJU, whereas no significant correlation was observed for HDS with these indicators. Notably, among these liver function indicators, only AST/HDL-C was significantly associated with MCI risk, while ZJU showed no significant association with cognitive outcomes. According to the 2021 Scientific Research Report on dietary guidelines for Chinese residents, the diet quality of most Chinese residents remains suboptimal. Notably, the median HDS for this population was only ‘1’ in this study, indicating that the per capita dietary intake was substantially below the recommendations of the Chinese Food Guide Pagoda (2022). Similarly, Chao et al. found that unbalanced dietary patterns significantly increase the risk of abnormal liver function (33). Thus, low HDS (MedianHDS = 1) could explain why this research’s correlation between diet and liver function was insignificant. However, liver function can be significantly improved through a healthy lifestyle, including abstaining from smoking and alcohol (25), drinking tea (27), performing housework, and engaging in physical activity (29).

We also explore the relationship between lifestyle and diet intake with liver and brain function. Patients with MCI had lower MoCA scores and shorter education duration. These findings are consistent with previous studies (34), suggesting the protective effect of good education on cognition. Regarding lifestyle, we observed that MCI patients were more likely to have drinking tea habits. The observed increased percentage of subjects with tea-drinking habits in MCI patients might be attributable to the health education of MCI patients for managing their lifestyle (27, 28). Although Sun et al. reported that excessive tea consumption could increase brain burden and might cause potential damage to brain function (35), most of the published documents emphasized the protective effect of moderate tea consumption on overall health status and the prevention of chronic diseases (27, 28), among them, Hu’s study based on the data of UK Biobank (36), found that consuming approximately three cups of tea per day (about 250 mL per cup, totaling ~750 mL/day) was associated with the greatest reduction in Alzheimer’s disease risk. In addition, we found that the subjects from the control group showed a greater preference for reading and housework. As a mental activity, reading was proved to significantly enhance cognitive function (37), while housework, as a form of moderate physical activity, was also found to influence cognition positively (29). Regarding liver function, we found that the MCI patients exhibited higher plasma AST/HDL-C ratios than the controls. This data further demonstrated the potential correlation between poorer liver function and the increased risk of cognitive impairment (4, 5).

We examined the relation between HDS, HLS, MoCA scores, and liver function indicators (AST/HDL-C, ALT/HDL-C, HSI, and ZJU). Our data demonstrated a significantly positive correlation between HDS and MoCA scores. Balance diets may alleviate cognitive impairment by promoting the hippocampus’s structural and functional plasticity and upregulating neurotrophic factors’ expression (38). These results suggest that a balanced dietary pattern may be critical in enhancing brain function. Zhang et al. found that brain regions were more sensitive to N-3 PUFA in diet than the liver (39). This conclusion also explains why diet might influence brain function more than liver function through the beneficial components of certain diets.

Interestingly, we observed a significant negative correlation between HLS and liver function indicators in the MCI population (Figure 2). This suggests that individuals with MCI may experience more significant improvements in liver function through adopting a healthy lifestyle. Whereas in the control group, a significant negative correlation was found between liver function indicators and cognitive scores. Aligning with the existing studies, Gao et al. advocated that early intervention in unhealthy livers could help prevent dementia (14). These data highlight the close association between impaired liver and cognitive decline. Furthermore, significant positive correlations among the four liver function indexes were also observed in our study, indicating their consistent and collective ability to reflect liver function.

We further analyzed the relation between HDS, HLS, liver function, and the risk of MCI. We did not observe a statistically significant association between HDS and the risk of MCI. However, adhering to a healthy lifestyle significantly reduces the risk of MCI. Regarding the relationship between liver function and cognition, after adjusting for confounders, elevated plasma AST/HDL-C and ALT/HDL-C ratios were associated with an increased risk of MCI. Previous studies have suggested that lower levels of liver enzyme ALT are linked to the increased brain Aβ protein deposition (40), while plasma HDL-C acts as a carrier of Aβ in the circulatory system to facilitate its metabolism (41). A decline in circulating HDL-C level may impair the body’s ability to clear circulatory Aβ. Thus, combining liver enzymes (AST and ALT) with plasma HDL-C provides better predictive capacity for MCI, likely reflecting their role in *in vivo* Aβ metabolism. This finding may also explain why HSI and ZJU, which are predictors of live function, did not show significant associations with the risk of MCI. Notably, the predictive power of AST/HDL-C and ALT/HDL-C for MCI underscores the connection between impaired liver function and cognitive decline. These results demonstrated the potential role of liver function-related markers in identifying individuals’ risk for MCI.

The results of RCS indicated that plasma AST/HDL-C and ALT/HDL-C ratios were positively associated with the risk of MCI, with cut-off values of 14.1 and 10.1, respectively. Prior studies have reported that the plasma AST/HDL-C and ALT/HDL-C ratio thresholds at 14.37 and 15.97 could be used to predict the damage status of liver function (11). As the most significant peripheral organ responsible for the clearance of Aβ, the liver plays a critical role in Aβ metabolism (42). The observed lower predictive thresholds for MCI than for hepatic dysfunction suggest that declining liver function may begin to impair cognition by reducing Aβ clearance even before liver function damage. Therefore, these data indicated the potential importance of maintaining normal liver function in inhibiting the pathology progress of cognitive decline.

This study examined the relationship between liver function, healthy lifestyle, dietary patterns, and cognitive function. Using the HLS and HDS, we comprehensively assessed participants’ lifestyles and dietary patterns. As expected, poor liver function was significantly associated with an increased risk of MCI. These findings highlight the importance of integrating lifestyle, dietary, and liver function considerations when interpreting cognitive outcomes in older adults. A healthy lifestyle and balanced diet demonstrated protective effects on both liver and cognitive functions, suggesting that interventions targeting these areas may help mitigate the risk of cognitive decline. Thus, beyond their individual effects, maintaining normal liver function alongside healthy lifestyle and diet patterns may be crucial in supporting cognitive health in the elderly.

The strength of our study is that, compared with conventional enzyme-based liver function evaluating indicators (such as AST, ALT, and GGT), the present study combined enzyme-based (AST and ALT) with non-enzyme-based parameter (HDL-C) to comprehensively evaluate individual’s liver function, which could more accurately reflect individual’s liver function status. This study has several limitations. Firstly, due to the cross-sectional nature of the data, we are unable to monitor dynamic changes in lifestyle, dietary patterns, liver function, and cognitive ability over time, which limits causal inference and the assessment of temporal relationships. Future research should employ longitudinal studies to address these gaps. Secondly, our data indicated that liver function might mediate the association between HLS, HDS, and cognition. The cross-sectional study design of the present study could not provide much more convincing proof. Thirdly, it should be noted that cognitive data of 630 participants were used for analyses. As a result, the sample size differed between analyses focusing on diet, lifestyle, and liver function and those including cognitive outcomes. This discrepancy reflects the use of the largest available dataset for each research focus rather than a comparison of different groups. Nevertheless, the exclusion of participants without cognitive assessments may introduce selection bias, and the generalizability of our findings regarding cognition should therefore be interpreted with caution. Therefore, a population-based cohort or interventional study is needed to further explore the underlying mechanisms.

Despite these limitations, our findings provide several practical implications for healthcare professionals and public health strategies. First, mental and physical activities, such as regular reading and engaging in housework, could be promoted as feasible lifestyle strategies to enhance cognitive resilience in older adults. Second, monitoring liver function markers such as the AST/HDL-C ratio may help identify individuals at elevated risk of cognitive decline, enabling earlier preventive interventions. Together, these recommendations highlight potential avenues for community-based programs aiming to reduce the burden of cognitive impairment.

## 5 Conclusion

Collectively, our findings indicated a strong association between liver and cognitive function, and AST/HDL-C could be a potential liver function-based parameter to predict the cognitive status of the elderly. Poor liver function correlates significantly with cognitive impairment, suggesting normal liver function is beneficial for the elderly to prevent cognitive function decline. Additionally, a healthy lifestyle and rationale dietary pattern might improve liver and brain function.

## Data Availability

The raw data supporting the conclusions of this article will be made available by the authors, without undue reservation.
