# Supplementary material for: Association of lifestyle, dietary pattern, and liver function with cognition in older adults: findings from a cross-sectional study
Source: Front Nutr. 2025 Sep 30;12:1655601. doi: 10.3389/fnut.2025.1655601 (PMC12518086; doi:10.3389/fnut.2025.1655601)
Supplement: Supplementary file 1 [file Table_1.DOCX]

Supplementary Material

# List of Abbreviations

| AD | Alzheimer’s disease |
| --- | --- |
| ALT | Alanine aminotransferase |
| AST | Aspartate transaminase |
| Aβ | β-amyloid |
| BBB | Blood-brain barrier |
| BMI | Body mass index |
| CV | Coefficient of variation |
| FFQ | Food frequency questionnaire |
| FLI | Fatty liver index |
| Glu | Glucose |
| HDL-C | High-density lipoprotein cholesterol |
| HDS | Healthy dietary score |
| HLS | Healthy lifestyle score |
| HSI | Hepatic steatosis index |
| LDL-C | Low-density lipoprotein cholesterol |
| NAFLD | Non-alcoholic fatty liver disease |
| MoCA | Montreal cognitive assessment |
| MCI | Mild cognitive impairment |
| NfL | Neurofilament light chain |
| RCS | Restricted cubic splines |
| ROC | Receiver operating characteristic curves |
| SD | Standard deviation |
| TC | Total cholesterol |
| TG | Triglycerides |
| T2DM | Type 2 diabetes mellitus |

# Supplementary Tables

Supplemental Table 1.The criteria for a healthy lifestyle score (HLS)

| Lifestyle category | Beneficial behavior | Assignment rule | Reference |
| --- | --- | --- | --- |
| Smoking | No | ‘1’ for the healthy behavior,  ‘0’ for the unhealthy behavior. | ^25,26^ |
| Alcohol drinking | No |  | ^25,26^ |
| Tea drinking | Yes |  | ^27,28^ |
| House working | Yes |  | ^29,30^ |
| Physical activity | Yes |  | ^29,30^ |

Supplemental Table 2. The criteria for a healthy diet score (HDS) according to Chinese Food Guide Pagoda (2022)

| Food Category | Recommended intake (g/d) | Assignment rule |
| --- | --- | --- |
| Cereal | 200 - 300 | ‘1’ for the intake within the recommended range, ‘0’ for outside the recommendation |
| Vegetable | 300 - 500 |  |
| Fruit | 200 - 350 |  |
| Animal food | 120 - 200 |  |
| Milk and dairy products | 300 - 500 |  |
| Soybeans and nuts | 25 - 35 |  |
| Cooking oil | 25 - 30 |  |

Supplemental Table 3. The predictors of non-alcoholic fatty liver disease (NAFLD)

| Indicators | Calculation formula | AUC | Reference |
| --- | --- | --- | --- |
| AST/HDL-C | AST (U/L) /HDL-C (mmol/L) | 0.787 | ^11^ |
| ALT/HDL-C | ALT (U/L) /HDL-C (mmol/L) | 0.855 |  |
| HSI | 8 × ALT (U/L) / AST (U/L)+BMI (kg/m^2^) (+2 if female, +2 if diabetes) | 0.812 | ^10^ |
| ZJU | BMI (kg/m^2^) + FPG (mmol/L) + TG (mmol/L) + 3 × ALT (U/L) / AST (U/L) (+2, if female) | 0.822 | ^31^ |

Supplemental Table 4. Demographic characteristics, daily dietary intakes, and plasma biochemical indices in total population (*N* = 1096)

| **Variables** | **All participants** |
| --- | --- |
| **Demographic characteristics** |  |
| Age, year (mean ± SD) | 69.21 ± 5.01 |
| Sex [ male (%)] | 600 (54.7) |
| BMI (kg/m²) | 24.27 ± 3.37 |
| T2DM (yes), n (%) | 365 (33.3) |
| **Lifestyle, n (%)** |  |
| Smoking [yes] | 208 (19.0) |
| Alcohol drinking [yes] | 253 (23.1) |
| Tea drinking [yes] | 412 (37.6) |
| House working [yes] | 913 (83.3) |
| Physical activity [yes] | 950 (86.7) |
| Reading [yes] | 274 (25.0) |
| TV and computer [yes] | 1004 (91.6) |
| **Dietary intake, (g/d)*** |  |
| Cereal | 275.00 (175.00, 325.00) |
| Vegetable | 275.00 (225.00, 375.00) |
| Fruit | 125.00 (75.00, 175.00) |
| Animal food | 119.64 (82.14, 166.07) |
| Soybean and nut | 32.14 (16.07, 71.43) |
| Milk | 107.14 (0.00, 196.43) |
| **Plasma biochemical indices (mmol/L)** |  |
| Glu | 5.90 (5.30, 8.60) |
| TC | 4.44 ± 1.61 |
| TG | 2.39 ± 1.97 |
| LDL-C | 2.31 ± 1.17 |
| HDL-C | 2.09 ± 1.27 |
| **Liver function** |  |
| AST (IU/L) | 19.00 (8.08, 24.00) |
| ALT (IU/L) | 14.00 (4.89, 21.00) |
| AST/HDL-C | 8.52 (5.82, 13.09) |
| ALT/HDL-C | 5.98 (3.43, 10.51) |
| HSI | 31.67 (28.30, 35.68) |
| ZJU | 36.79 (32.65, 43.40) |
| **Comprehensive evaluation** |  |
| HDS | 1.00 (1.00, 2.00) |
| HLS | 4.00 (3.00, 4.00) |

The data were expressed as mean±SD or n (%) according to whether the continuous or category variable, respectively. BMI, body mass index; T2DM, type 2 diabetes mellitus; Glu, glucose; TC, total cholesterol; TG, triglyceride; LDL-C, low-density lipoprotein cholesterol; HDL-C, high-density lipoprotein cholesterol; AST, aspartate transaminase; ALT, alanine aminotransferase; HSI, hepatic steatosis index; HDS, healthy diet score; HLS, healthy lifestyle score. * The information of daily cooking oil consumption is incomplete, it has not been analyzed or included in the HDS calculation.

# Supplementary Figures

**Supplemental Figure 1. The study flowchart of participants recruitment.**
