# Supplementary figures and images for: Association of lifestyle, dietary pattern, and liver function with cognition in older adults: findings from a cross-sectional study
Source: Front Nutr. 2025 Sep 30;12:1655601. doi: 10.3389/fnut.2025.1655601 (PMC12518086; doi:10.3389/fnut.2025.1655601)

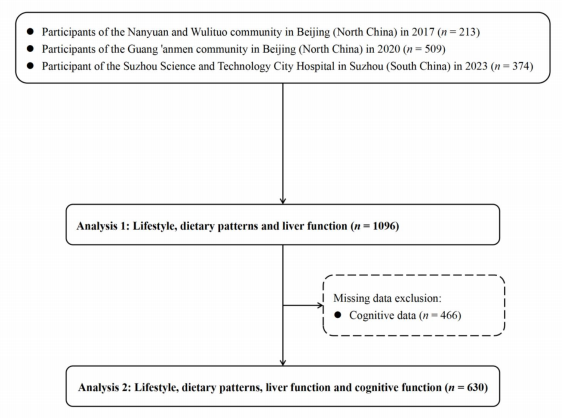

Supplement: Supplementary file 2 [file Image_1.TIF]
